# Cost-effectiveness analysis of treatment of venous thromboembolism with rivaroxaban compared with combined low molecular weight heparin/vitamin K antagonist

**DOI:** 10.1186/s12959-015-0051-3

**Published:** 2015-06-11

**Authors:** Luke Bamber, Dominic Muston, Euan McLeod, Anne Guillermin, Julia Lowin, Raj Patel

**Affiliations:** Bayer Pharma AG, Wuppertal, Germany; Bayer HealthCare Pharmaceuticals Inc., Whippany, NJ USA; IMS Health, London, UK; Department of Haematological Medicine, King’s College Hospital, London, UK

**Keywords:** Cost-effectiveness, Rivaroxaban, Venous thromboembolism treatment

## Abstract

**Background:**

Venous thromboembolism (VTE) is a burden on healthcare systems. Standard treatment involves parenteral anticoagulation overlapping with a vitamin K antagonist, an approach that is effective but associated with limitations including the need for frequent coagulation monitoring. The direct oral anticoagulant rivaroxaban is similarly effective to standard therapy as a single-drug treatment for VTE and does not require routine coagulation monitoring. The objective of this economic evaluation was to estimate the cost-effectiveness of rivaroxaban compared with standard VTE treatment from a UK perspective.

**Methods:**

A Markov model was constructed using data and probabilities derived from the EINSTEIN DVT and EINSTEIN PE studies of rivaroxaban and other published sources. Health outcomes included VTE rates, bleeding events avoided, quality-adjusted life-years (QALYs) and incremental cost-effectiveness ratios (ICERs).

**Results:**

There was greater discounted quality-adjusted life expectancy with rivaroxaban than with standard therapy, irrespective of indication and treatment duration. Rivaroxaban was associated with per-patient cost savings for each treatment duration modelled (3, 6 and 12 months), and these were greatest with shorter durations. Rivaroxaban was found to be dominant (cheaper and more effective) and, therefore, cost-effective, in both patients with deep vein thrombosis and pulmonary embolism in all three treatment duration groups, and was also cost-effective in patients requiring lifelong anticoagulation (ICERs: £8677 per QALY and £7072 per QALY in patients with index deep vein thrombosis and pulmonary embolism, respectively). The cost-effectiveness of rivaroxaban was largely insensitive to variations in one-way sensitivity analysis. Probabilistic sensitivity analysis demonstrated that at a threshold of £20,000 per QALY, rivaroxaban had a consistent probability of being cost-effective, compared with LMWH/VKA treatment, of around 80% regardless of index VTE or duration of anticoagulation therapy (3, 6, 12 months or lifelong).

**Conclusions:**

This analysis suggests that rivaroxaban represents a cost-effective choice for acute treatment of deep vein thrombosis and pulmonary embolism and secondary prevention of VTE in the UK, compared with LMWH/VKA treatment, regardless of the required treatment duration.

**Electronic supplementary material:**

The online version of this article (doi:10.1186/s12959-015-0051-3) contains supplementary material, which is available to authorized users.

## Background

Venous thromboembolism (VTE) is both an acute, potentially life-threatening and chronic condition that continues to present a burden to healthcare systems [1-3]. The annual incidence of VTE, which includes deep vein thrombosis (DVT) and pulmonary embolism (PE), has been estimated at 1–2 cases per 1000 persons per year in western populations [4-6] as well as in the UK [7]. Long-term sequelae of VTE include recurrent thromboembolism, post-thrombotic syndrome (PTS) and chronic thromboembolic pulmonary hypertension (CTEPH) [8,9]. These serious complications further add to the burden of managing VTE [10].

In the UK, treatment of acute DVT and PE and prevention of recurrent VTE primarily involve the use of anticoagulant therapy. Current standard of care consists of initial treatment with a parenteral anticoagulant, often low molecular weight heparin (LMWH; administered by subcutaneous injection) overlapping with a vitamin K antagonist (VKA), typically warfarin [11-13]. However, VKAs have various limitations, including a narrow therapeutic range, a requirement for dose adjustment, a response that is easily influenced by diet and concomitant medication, and a requirement for frequent international normalized ratio (INR) monitoring [14]. The typical ongoing frequency of monitoring in the UK is every 3–4 weeks, but it can be even more frequent, particularly during initiation of treatment [15-18]. The objective of monitoring is to maintain the INR level within the therapeutic range to prevent the recurrence of VTE while minimizing the increased risk of bleeding. The resulting need for routine collection of blood samples and subsequent laboratory analysis can make the use of VKAs inconvenient for patients and imposes a cost and management burden on healthcare systems [19-21]. LMWHs are also associated with safety issues, particularly when used without careful individualized dosing [22], and furthermore, patients with poor dexterity may experience difficulties with subcutaneous self-administration.

There is no clear consensus on the optimal duration of anticoagulant treatment in the UK; however, treatment is generally recommended for at least 3 months after the venous thromboembolic event [15,23], and then possibly extended with regular reassessments [12]. In a retrospective observational study, variations in the duration of anticoagulation treatment were observed in UK anticoagulation treatment practice, confirming the relevance of the EINSTEIN trial approach of comparing across patient populations requiring different durations of treatment [24].

The EINSTEIN DVT and EINSTEIN PE studies were designed to investigate the efficacy and safety of rivaroxaban compared with standard therapy in the treatment of patients with confirmed acute, symptomatic DVT or PE, respectively [25,26]. In both trials, patients were randomized in an open-label, event-driven, non-inferiority study design that compared 3 weeks of rivaroxaban 15 mg twice daily followed by 20 mg once daily for an additional 3, 6 or 12 months, with standard dual-drug therapy (LMWH [enoxaparin] followed by a VKA) [25,26]. The duration of therapy was selected by the treating clinician to reflect local preferences, the underlying risks arising from the nature of the index VTE, characteristics of the patient, and ongoing risk of recurrent VTE or bleeding [25,26].

Results of both studies showed non-inferiority of rivaroxaban for the primary efficacy endpoint (the composite of recurrent DVT and fatal and non-fatal PE) compared with the current standard therapy [25,26]. In a pooled analysis of the two studies, rivaroxaban showed a similar incidence to standard therapy for the principal safety outcome of major and non-major clinically relevant bleeding; there was also a significant reduction in the incidence of major bleeding with rivaroxaban (hazard ratio 0.54; 95% confidence interval [CI] 0.37–0.79; p = 0.002) [27].

Although the drug acquisition costs of rivaroxaban may be higher than those associated with LMWH/VKA therapy, it is possible that rivaroxaban therapy may be more cost-effective, owing to the complexities of VKA therapy, particularly the frequent INR monitoring. Rivaroxaban has been shown to be cost-effective for the prevention of VTE compared with enoxaparin in a UK model [28]. However, published data surrounding the potential cost-effectiveness of rivaroxaban in VTE treatment are scarce. The objective of this economic evaluation was to estimate, from the perspective of the UK National Health Service (NHS), the cost-effectiveness of rivaroxaban compared with standard care in the treatment of acute DVT or PE based on the findings of the EINSTEIN DVT and EINSTEIN PE studies. The model presented here provided the basis for the health economic evidence submissions made to the UK National Institute for Health and Care Excellence (NICE) for the appraisal of rivaroxaban for the treatment of DVT and PE [29,30].

## Methods

### Model structure

**Figure 1:**
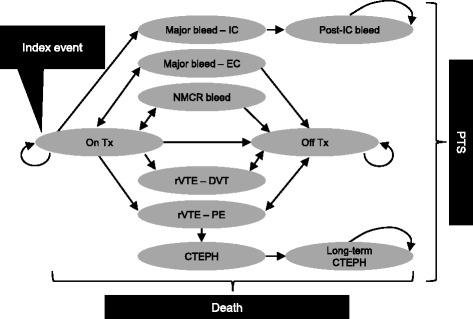
State diagram for the economic model. The model states referred to here are described in Table 1. Note that states, On Tx-post DVT, and, PE post DVT, were specific to analyses for patients post index PE and have been omitted from the diagram. PTS risk was restricted to states with a DVT history in the PE analysis. CTEPH, chronic thromboembolic pulmonary hypertension; DVT, deep vein thrombosis; EC, extracranial; IC, intracranial; NMCR, non-major clinically relevant; PE, pulmonary embolism; PTS, post-thrombotic syndrome; rVTE, recurrent venous thromboembolic event; Tx, treatment.

A Markov model design was selected after a review of the existing cost-effectiveness literature and based on consultation with UK clinical and health economic experts [31-34]. Patients entered the model after a diagnosis of VTE; these patients were categorized according to whether they had experienced an index DVT or PE. Patient progression between model health states was according to 3-month cycles and transition probabilities derived from the EINSTEIN clinical trials or published studies. The model time horizon was 40 years, approximating to a lifetime for this patient group. The health states described the health outcomes and resource implications of initial VTE management as well as potential complications (Table 1 and Figure 1).

**Table 1:** **Descriptions of the health states in the DVT and PE models**

**State name**	**Description**
On Tx	Patients who have just experienced an acute VTE, and are receiving one of the acute treatments being evaluated (either 3, 6 or 12 months or lifetime treatment with rivaroxaban or dual LMWH/VKA therapy)
rVTE – DVT	Patients who have just experienced a recurrent DVT. Assigned therapy was discontinued and all patients assumed to receive 6 months of dual LMWH/VKA. The duration of utility impact was assumed to be 1 month in the base case. DVT events were not associated with excess mortality
rVTE – PE	Patients who have just experienced a recurrent PE (± DVT). Patients with coincident DVT transit to a post-DVT state to capture PTS risk. Assigned therapy was discontinued and all patients assumed to receive 6 months of dual LMWH/VKA. The duration of utility impact was assumed to be 1 month in the base case. PE events were associated with excess mortality
Major bleed – IC	Patients on assigned therapy who have just experienced an IC bleeding event. Therapy was temporarily withheld during the cycle in which the IC bleeding event took place. IC bleeding events were associated with excess mortality
Major bleed – EC	Patients on assigned therapy who have just experienced a major EC bleeding event (e.g. gastrointestinal bleeding). Therapy was temporarily withheld for 1 month during the cycle in which the bleeding event took place. The duration of utility impact was assumed to be 1 month in the base case
NMCR bleed	Patients on assigned therapy who have just experienced a NMCR bleeding event. Defined as overt bleeding that did not meet the criteria for major bleeding but was associated with medical intervention, unscheduled contact with a physician, interruption or discontinuation of a study drug, or discomfort or impairment of activities of daily life. Therapy was temporarily withheld for 1 month during the cycle in which the bleeding event took place. An example of this would be spontaneous bleeding from gums which requires acute medical intervention. NMCR bleeding was assumed not to impact on utility
Post-IC bleed	Patients who previously experienced an IC bleeding event. Any assigned therapy is assumed to stop. IC bleeding events are associated with major risks of residual disability stemming from their impact on the central nervous system. The health-related quality of life and costs associated with this are included
Off Tx-post index PE*	Patients currently off treatment after index PE. These patients are not at ongoing risk of PTS
Off Tx-post DVT	Patients who have experienced an incident DVT within the time frame of the model and who are currently off treatment. These patients are at risk of PTS
On Tx-post DVT	This state is only applicable to analyses of lifelong treatment duration. Patients who have experienced an incident DVT within the time frame of the model and who are currently on treatment. These patients are at risk of PTS
PE post DVT*	Patients with recurrent PE and a history of DVT within the model. Survivors return to relevant post-DVT states so as to continue exposure to a risk of PTS conferred by their DVT history
CTEPH	Patients diagnosed with CTEPH who are exposed to management costs, health-related quality of life loss and excess mortality
Long-term CTEPH	State to which patients with CTEPH transition in the long term
Death	Terminal state. Patients could die because of either events captured in the model, such as PE or IC bleed, or from other causes

*PE model-specific health states.

CTEPH, chronic thromboembolic pulmonary hypertension; DVT, deep vein thrombosis; EC, extracranial; IC, intracranial; LMWH, low molecular weight heparin; NMCR, non-major clinically relevant; PE, pulmonary embolism; PTS, post-thrombotic syndrome; rVTE, recurrent venous thromboembolism; Tx, treatment; VKA, vitamin K antagonist; VTE, venous thromboembolism.

The analysis was undertaken from a UK payer perspective (NHS and personal social services) in accordance with the NICE Reference Case [35]. An annual discount rate equal to 3.5% was adopted for costs and outcomes [35]. Health outcomes modelled included numbers and rates of venous thromboembolic events, bleeding events avoided, quality-adjusted life-years (QALYs) and economic outcomes such as costs and incremental cost-effectiveness ratios (ICERs).

### Patient population and treatment

Patients entering the model mirrored the inclusion criteria for the EINSTEIN DVT and EINSTEIN PE studies and had a mean age of 56 and 58 years at baseline, respectively [25,26]. The duration of rivaroxaban or standard therapy (LMWH/VKA) – 3, 6 or 12 months – was determined according to each patient’s risk profile. Rivaroxaban was administered according to its subsequently licenced schedule for DVT and PE treatment: 15 mg twice daily for 21 days followed by 20 mg once daily for the remaining duration of anticoagulation treatment [36]. For dual-drug therapy with LMWH/VKA, LMWH was continued until therapeutic anticoagulation with a VKA had been established [37]. Based on the study design of EINSTEIN DVT and EINSTEIN PE [25,26], and the pattern of LMWH use in the UK, enoxaparin was selected as the LMWH for the model. The daily dose of enoxaparin was 1.5 mg/kg, in alignment with the regimen licensed for the UK [12]. The LMWH/VKA comparator with varying treatment durations selected for the model was in concordance with guidelines [15,23] and observed practice in the UK [24]. A scenario evaluating patients with persisting risk of VTE requiring lifelong anticoagulation was included.

### Model input data

#### Clinical parameters and variables

For patients receiving dual-drug therapy with LMWH/VKA, the baseline incidences of recurrent VTE, major bleeding and non-major clinically relevant bleeding were derived from EINSTEIN DVT and EINSTEIN PE [25,26]. These were evaluated according to intended treatment duration to account for differences in patient risk profiles (Table 2). Transition probabilities during the on-treatment period were also differentiated according to time since the index event, to account for changes in VTE risk over time. The effect of rivaroxaban was assumed to apply while a patient was on treatment. Treatment effects were applied as either hazard ratios or relative risks and taken from either EINSTEIN DVT or EINSTEIN PE, depending on the index event for the cohort. Consistent with the NICE Reference Case, treatment effects were applied regardless of significance [35].

**Table 2:** **Incidence of clinical events in EINSTEIN DVT** [25] **and EINSTEIN PE** [26]

**Probability, mean (SE)**	**EINSTEIN DVT**	**EINSTEIN PE**
**Recurrent VTE (LMWH/VKA)**		
**3-month population**		
0–3 months	0.015 (0.008)	0.016 (0.011)
**6-month population**		
0–3 months	0.024 (0.005)	0.016 (0.003)
3–6 months	0.003 (0.002)	0.002 (0.001)
**12-month population**		
0–3 months	0.035 (0.009)	0.015 (0.004)
3–6 months	0.008 (0.004)	0.003 (0.002)
6–12 months	0.003 (0.003)	0.001 (0.001)
**Lifelong population** [61]		
>12 months	0.700 (0.107)	0.700 (0.107)
**Major bleeding (LMWH/VKA)**		
**3-month population**		
0–3 months	0.020 (0.010)	0.041 (0.018)
**6-month population**		
0–3 months	0.009 (0.003)	0.010 (0.003)
3–6 months	0.004 (0.002)	0.008 (0.003)
**12-month population**		
0–3 months	0.002 (0.002)	0.013 (0.004)
3–6 months	– (0.002)	0.004 (0.002)
6–12 months	– (0.002)	0.006 (0.003)
**Lifelong population** [61]		
>12 months	1.600 (0.245)	1.600 (0.245)
**NMCR bleeding (LMWH/VKA)**		
**3-month population**		
0–3 months	0.060 (0.017)	0.066 (0.022)
**6-month population**		
0–3 months	0.047 (0.006)	0.067 (0.007)
3–6 months	0.013 (0.004)	0.022 (0.004)
**12-month population**		
0–3 months	0.049 (0.01)	0.062 (0.008)
3–6 months	0.024 (0.008)	0.029 (0.006)
6–12 months	0.038 (0.01)	0.030 (0.006)
**Lifelong population**		
>12 months	0.014 (0.002)	0.022 (0.002)

LMWH, low molecular weight heparin; NMCR, non-major clinically relevant; SE standard error; VKA, vitamin K antagonist; VTE, venous thromboembolism.

Composite outcomes were disambiguated according to the observed split in each trial (Table 3). Additionally, for the PE cohort, the model considered the proportion of recurrent PE patients with concurrent DVT (3.4%), to account for differences in long-term risk of PTS.

**Table 3:** **Overview of assumed clinical parameters for DVT and/or PE patients**

**Probability, mean (SE)**	**DVT patients**	**PE patients**	**Source**
**Event/outcome**			
Incidence of recurrent VTE (HR)	0.68 (0.218)	1.123 (0.207)	*
Incidence of major bleeding (HR)	0.646 (0.242)	0.493 (0.24)	*
Incidence of NMCR bleeding (RR)	1.055 (0.123)	1.001 (0.088)	*
Probability that a recurrent VTE is a DVT	0.483 (0.054)	0.372 (0.050)	*
Probability that a major bleeding event is a (major) IC bleed	0.125 (0.058)	0.143 (0.076)	*
**Discontinuation**			
Patients with IC bleeding events	1.00 (0.0)	1.00 (0.0)	*
Patients with major EC bleeding events	0.400 (0.089)	0.164 (0.045)	*
Patients with NMCR bleeding events	0.110 (0.020)	0.054 (0.010)	*
For any other reason (additional) 3–12 months	0.019 (0.001)	0.021 (0.001)	*
For any other reason (additional) >12 months	0.036 (0.013)	0.036 (0.013)	Boggon 2011 [62]
	**DVT and PE patients**		
**Risks of subsequent morbidities**			
Recurrent VTE (per 3-month time step)	0.013 (0.074)		Prandoni 2007 [41]
Progression to CTEPH after a PE	0.013 (0.002)		Miniati 2006 [38]
Cumulative incidence of severe PTS (to 1 year)	0.027 (0.007)		Prandoni 1997 [40]
Cumulative incidence of severe PTS (to 5 years)	0.081 (0.012)		Prandoni 1997 [40]
**Mortality associated with another model event**			
PE (during acute treatment phase)	0.250 (0.041)		*
PE (after acute treatment phase)	0.331 (0.041)		Prandoni 1997 [40]
Major IC bleeding	0.436 (0.036)		Linkins 2010 [42]
Major EC bleeding	0.039 (0.007)		*
CTEPH (per 3-month cycle)	0.025 (0.020)		Condliffe 2008 [43]

*Data are from EINSTEIN DVT or EINSTEIN PE unless otherwise stated [25,26].

CTEPH, chronic thromboembolic pulmonary hypertension; DVT, deep vein thrombosis; EC, extracranial; HR, hazard ratio; IC, intracranial; NMCR, non-major clinically relevant; PE, pulmonary embolism; PTS, post-thrombotic syndrome; RR, relative risk; SE, standard error; VTE, venous thromboembolism.

Longer-term implications of anticoagulant treatment were estimated based on published data identified in a systematic literature review (Table 3) [25,26,38-43]. Based on the index event, patients were exposed to either a long-term risk of PTS (post DVT) or CTEPH (post PE). The additional risk of either PTS or CTEPH was captured for recurrent VTE. The risk of recurrent VTE while off treatment was based on long-term observational research [41].

The risk of mortality comprised two parts: the age-associated risk for the patient population, sourced from UK life tables from the Office of National Statistics (www.ons.gov.uk), and the risk of mortality associated with particular model events. Events associated with additional mortality included: PE during both the acute treatment and secondary prevention phase, PE after the treatment phase, major intracranial bleeding, major extracranial bleeding, and CTEPH. These risks were informed by systematic literature review (Table 3). With the exception of the ongoing risk for mortality from CTEPH, each mortality risk was applied once in the model, at the time of the relevant event.

#### Utility values

Evidence of utility values associated with VTE, including events such as DVT, PE, bleeding, CTEPH and PTS, in patient populations with VTE, was sought through systematic literature review [44-47]. The review also set out to identify evidence that might suggest moderation of utilities according to the nature of treatment received. The starting point in the modelling of utility in the pharmacoeconomic evaluation was the population norm value of 0.825 established in the landmark national EQ-5D survey [48], which was used as an anchor point in this evaluation. The utility values adopted for the cost-effectiveness analyses are summarized in Table 4.

**Table 4:** **Utility values assumed in the cost-effectiveness evaluation**

**Model state**	**Mean**	**Sensitivity analyses**		**Source**
		**Lower**	**Upper**	
Population norm	0.825	0.819	0.831	Kind 1998 [48]
Post-IC bleeding	0.71	0.70	0.72	Rivero-Arias 2010 [44]
CTEPH	0.56	0.53	0.59	Meads 2008 [45]
**Adjustments to utility norm due to modelled events**				
DVT	0.84	0.64	0.98	Locadia 2004 [46]
PE	0.63	0.36	0.86	Locadia 2004 [46]
EC bleeding (gastrointestinal bleeding was the disease state valued)	0.65	0.49	0.86	Locadia 2004 [46]
IC bleeding (haemorrhagic stroke was the disease state valued)	0.33	0.14	0.53	Locadia 2004 [46]
PTS (serious PTS was the disease state valued)	0.93	0.91	1.00	Lenert 1997 [47]

Locadia *et al*. quoted a population norm (own health) as 0.95 (95% confidence interval [CI] 0.81–1.00) [46]. Utility values were adjusted according to this value before adjusting for UK population norm.

Lower and upper values are estimates of 95% CIs from data presented (e.g. sample population size, n and standard deviation) in the source literature.

The 95% CIs for DVT, PE, and EC and IC bleeding adjustments to utility norms have been assumed to equal the interquartile range because of the absence of further information and the size of the sample in Locadia *et al.* [46].

For the probabilistic sensitivity analyses, the parameters above were modelled as arising from independent beta distributions with alpha and beta parameters set such that the mean is the point estimate and the lower and upper values represent the 95% CI.

CTEPH, chronic thromboembolic pulmonary hypertension; DVT, deep vein thrombosis; EC, extracranial; IC, intracranial; PE, pulmonary embolism; PTS, post-thrombotic syndrome.

The most appropriate evidence on the disutility for DVT, PE, major extracranial bleeding (assumed to match the disutility reported for gastrointestinal bleeding) and intracranial bleeding (assumed to match the disutility report for haemorrhagic stroke) was an evaluation of patient preferences in VTE [46]. Treatment satisfaction with rivaroxaban has been shown to be higher than with LMWH/VKA in the EINSTEIN studies [49,50]. Therefore, it was reasonable to assume that VKA treatment was associated with a disutility. All other utility assumptions were made independent of treatment arm.

#### Cost and resource input data

The model includes resource consumption related to the index event with respect to drug acquisition components and associated monitoring requirements (Table 5). Patients in the LMWH/VKA arm of the model initially require acute treatment with LMWH, overlapping with and followed by a VKA.

**Table 5:** **Base case results**

	**EINSTEIN DVT**			**EINSTEIN PE**		
	**Rivaroxaban**	**Dual LMWH/VKA therapy**	**Incremental**	**Rivaroxaban**	**Dual LMWH/VKA therapy**	**Incremental**
**Patients appropriate for 3 months of anticoagulation**						
Drug acquisition cost (£)	220	98	122	217	99	118
Other costs (£)	1592	1964	−372	4295	4808	−513
Total costs (£)	1812	2063	−251	4511	4907	−396
QALYs	13.286	13.264	0.022	11.940	11.912	0.027
ICER (£)			Rivaroxaban dominates			Rivaroxaban dominates
**Patients appropriate for 6 months of anticoagulation**						
Drug acquisition cost (£)	398	104	295	393	105	288
Other costs (£)	1561	2040	−479	4153	4654	−501
Total costs (£)	1959	2143	−184	4546	4759	−213
QALYs	13.294	13.268	0.026	11.992	11.979	0.013
ICER (£)			Rivaroxaban dominates			Rivaroxaban dominates
**Patients appropriate for 12 months of anticoagulation**						
Drug acquisition cost (£)	731	114	617	728	115	613
Other costs (£)	1512	2186	−673	4154	4900	−746
Total costs (£)	2243	2299	−56	4881	5015	−133
QALYs	13.308	13.274	0.034	12.035	12.015	0.020
ICER (£)			Rivaroxaban dominates			Rivaroxaban dominates
**Patients appropriate for lifelong anticoagulation**						
Drug acquisition cost (£)	6566	288	6278	6025	284	5740
Other costs (£)	2084	6835	−4751	4532	9209	−4677
Total costs (£)	8649	7122	1527	10,557	9493	1064
QALYs	13.507	13.331	0.176	12.526	12.375	0.150
ICER (£)			8677			7072

ICER, incremental cost-effectiveness ratio; LMWH, low molecular weight heparin; QALY, quality-adjusted life-year; VKA, vitamin K antagonist.

Because warfarin is the most commonly used VKA for VTE treatment in the UK [12], and was also the most frequently used VKA in the EINSTEIN DVT and EINSTEIN PE studies [25,26], it was selected for the model. To facilitate understanding of the contemporary provision of anticoagulation care, a survey of models of care was conducted (Bayer, data on file) [51]. Results suggested that, instead of traditional secondary care (consultant-led services), primary care is now the most common setting for provision of these services. Therefore, based on the results of this survey, the primary care-setting cost was applied in 66.5% of cases and the secondary care-setting cost in the remaining 33.5% of cases; transport services were required by 8.6% of patients managed in secondary care. It was assumed that patients receiving a VKA would require a mean of nine coagulation monitoring visits in the first 3 months and five visits per quarter thereafter (Table 6) [15,18,25,51].

**Table 6:** **Summary of resource usage assumptions**

**Resource item**	**Mean**	**Sensitivity analyses**			**Rationale**
		**Lower**	**Upper**	**Distribution**	
**Acute treatment**					
Number of days of acute treatment (i.e. LMWH) required by a DVT patient	9.6	6	10	Dirichlet	EINSTEIN DVT [25]
					SIGN guidelines [15]
Number of days of acute treatment (i.e. LMWH) required by a PE patient	9.7	7	13	Dirichlet	Mean duration from EINSTEIN PE [26]
Proportion of patients who self-inject LMWH (%)	92	64.40	100	Beta	The point estimate is taken from the assumptions in NICE CG92 [56]. The sensitivity range is an assumption
Proportion of remaining patients who require nurse assistance at home (%)	80	60	100%	Beta	These values are assumptions based on inputs determined for the NICE CG92 model
**INR monitoring while on LMWH/VKA**					
Visits in first 3 months	9	5	15	Gamma	EINSTEIN DVT [25,51]
					SIGN guidelines [15,51]
Visits each 3 months thereafter	5	2.5	10	Gamma	BNF [18,51]
**Recurrent VTE: proportion treated as outpatients rather than inpatients**					
Recurrent DVT patients (%)	69	50	100	Beta	SIGN guidelines [15]
Incident PE patients (%)	17	0	30	Beta	Survey data
**Other**					
Proportion of patients requiring NHS-funded transportation (%)	8.55	6	11	Beta	Survey data
Proportion of CTEPH patients who require PEA (%)	68.40	64.20	72.60	Beta	321 of 469 patients from Condliffe 2008 [43]
Length of admission post DVT, days					van Bellen 2014 [52]
LMWH/VKA	8*	4	10		
Rivaroxaban	5*	3	9		
Length of admission post PE, days					van Bellen 2014 [52]
LMWH/VKA	7*	5	10		
Rivaroxaban	6*	4	9		

*Median values.

BNF, British National Formulary; CTEPH, chronic thromboembolic pulmonary hypertension; DVT, deep vein thrombosis; INR, international normalized ratio; LMWH, low molecular weight heparin; NHS, National Health Service; NICE, National Institute for Health and Care Excellence; PE, pulmonary embolism; PEA, pulmonary endarterectomy; SIGN, Scottish Intercollegiate Guidelines Network; VKA, vitamin K antagonist; VTE, venous thromboembolism.

In addition, both the EINSTEIN DVT and EINSTEIN PE studies demonstrated that hospitalized patients treated with rivaroxaban had significantly shorter durations of admission than patients treated with LMWH/VKA [52]. Savings related to this for the index event were included in the analyses. The economic model also accounted for resource usage associated with bleeding (of various types/severities), recurrent VTE, PTS and CTEPH (Table 6).

#### Unit costs

Unit costs used in the model were taken wherever possible from the NHS National Schedule of Reference Costs, the Personal Social Services Research Unit and the British National Formulary [18,53,54]. All unit costs assumed in the economic model are listed in Additional file 1, together with the source and rationale for each value [18,53-58].

### Analyses performed

The base case evaluated the costs and health outcomes of rivaroxaban compared with dual LMWH/VKA therapy among patients who required anticoagulation therapy for 3, 6 or 12 months, reflecting the design of EINSTEIN DVT and EINSTEIN PE. In addition, a scenario analysis was undertaken comparing rivaroxaban with LMWH/VKA for patients requiring lifelong anticoagulation.

### Sensitivity analyses

To identify key model drivers, all inputs were subject to univariate sensitivity analyses. Clinical parameters were varied between 95% CIs. Utility value assumptions were varied between 95% CIs or, where these were not available, the interquartile range. Mean age at baseline was varied between 45 and 75 years. Resource usage was varied as described in Table 6 and unit cost values were varied by interquartile ranges. The setting of INR monitoring varied from a mixture of primary and secondary care, as described previously, to either 100% primary or 100% secondary care.

Probabilistic sensitivity analyses were conducted to estimate the effect of overall uncertainty in the economic evaluation through repeated sampling of mean parameter values from a series of assigned distribution types. The analyses were used to generate cost-effectiveness planes and cost-effectiveness acceptability curves across patients requiring different treatment durations. The type of distribution applied to a given parameter was dependent on the nature of that parameter (Tables 1, 2, 3, 4, 5 and 6).

## Results

Results are presented according to the index event and appropriate duration of anticoagulation therapy (Table 5). There was greater discounted quality-adjusted life expectancy with rivaroxaban than with LMWH/VKA, irrespective of indication and treatment duration. Rivaroxaban was associated with per-patient cost savings for each of the trial-based treatment durations (3, 6 and 12 months). Savings were greatest with shorter treatment durations. As a consequence, rivaroxaban was found to be dominant (cheaper and more effective) and, therefore, cost-effective, in both DVT and PE patients in all three treatment duration groups. Rivaroxaban was also cost-effective in the scenario analysis of patients requiring lifelong anticoagulation, with ICERs of £8677 per QALY and £7072 per QALY in patients with index DVT and PE, respectively.

### Sensitivity analyses

**Figure 2:**
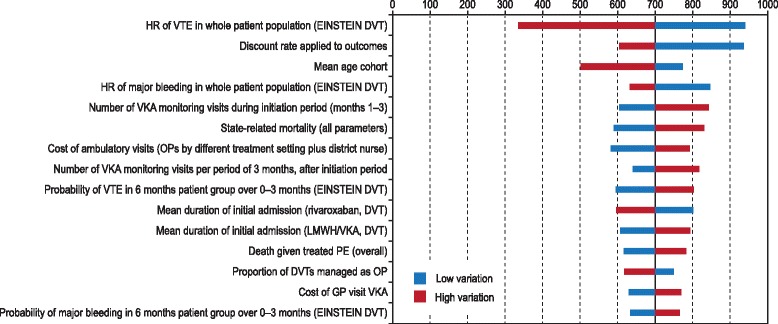
Tornado diagram of net monetary benefit of rivaroxaban versus LMWH/VKA. Patients requiring 6 months of anticoagulation. DVT, deep vein thrombosis; GP, general practitioner; HR, hazard ratio; LMWH, low molecular weight heparin; OP, outpatient; OWSA, one-way sensitivity analysis; PE, pulmonary embolism; QALY, quality-adjusted life-year; VKA, vitamin K antagonist; VTE, venous thromboembolism.

Overall, more than 150 sensitivity analyses were conducted for each of the patient groups evaluated. The cost-effectiveness of rivaroxaban versus LMWH/VKA was largely insensitive to variation in the assumptions made. Notably, findings were stable when either savings associated with earlier discharge in rivaroxaban patients or warfarin disutility was removed. Sensitivity was greatest to treatment effects and the frequency and cost of INR monitoring, and an illustrative tornado plot for the 15 most sensitive parameters in the 6-month treatment duration for DVT is provided in Figure 2 (Please see Additional file 1: Figure S1 and Additional file 2: Figure S2 for the complete set of parameters for 3, 6, 12 and lifelong treatment duration for DVT and PE). Results are shown using the Net Monetary Benefit measure at a willingness to pay of £20,000 per QALY because presenting ICER results was less meaningful owing to the dominance of rivaroxaban.

The probabilistic sensitivity analyses demonstrated that at a threshold of £20,000 per QALY, the probability of rivaroxaban being cost-effective in comparison with LMWH/VKA was greater than 81%, regardless of index VTE or whether a patient required 3, 6 or 12 months of anticoagulation therapy. For patients requiring lifelong anticoagulation, the likelihood was 80% and 78%, for DVT and PE patients, respectively.

## Discussion

The current analyses evaluated the cost-effectiveness of rivaroxaban relative to dual LMWH/VKA therapy for the treatment of VTE and associated complications. For patients requiring up to 12 months of anticoagulant treatment, rivaroxaban was associated with lower costs and greater QALYs than dual LMWH/VKA therapy and, therefore, dominated the current standard of care (dual-drug therapy with LMWH/VKA) in the base case. For patients requiring lifelong anticoagulation, rivaroxaban had an ICER of below £9000 per QALY gained; therefore, rivaroxaban can be considered cost-effective according to commonly accepted UK willingness-to-pay thresholds. Sensitivity analysis established that these findings were robust to input variation.

Several factors were identified as drivers of results. Cost-effectiveness was found to be driven by improved safety, in terms of fewer major bleeding events, leading to a higher number of QALYs gained. Greater cost savings and increased incremental QALYs for rivaroxaban were associated with groups of patients requiring shorter durations of therapy and for whom earlier discharge is feasible.

A potential drawback of rivaroxaban, as with the other novel anticoagulants, is the lack of a specific antidote. The impact of this limitation on the cost-effectiveness model would be mediated through either increased cost of bleeding management or poorer patient outcomes in comparison with VKAs, for which a slow acting antidote exists. However, evidence from the pooled EINSTEIN programme data on the clinical impact of bleeding suggested that major bleeding in the rivaroxaban arm had a milder clinical presentation, and took a milder course, than LMWH/VKA treatment [59]. Based on this evidence, no differential assumptions on event costs or clinical outcomes after a bleeding event have been applied. The availability of a specific reversal agent could potentially improve the existing safety profile of rivaroxaban [60], providing a welcome additional treatment option in case of life-threatening bleeding, and further clinical reassurance. However, the impact on overall cost-effectiveness would be limited.

### Strengths and limitations

As with any economic evaluation, the current analyses encompassed a number of strengths and limitations. An area of weakness of the model is that there is no single source for the resource use estimates, including frequency of monitoring visits, proportion of patients who require nursing assistance with injection and cost of transportation to the healthcare facility for anticoagulation monitoring. These assumptions were taken from the results of literature searches and represent the best available sources. However, the impact of rivaroxaban will vary with local conditions, and this should be considered in the interpretation of the results. A particularly critical area of uncertainty identified *a priori* during model development was INR monitoring frequency. Owing to wide variation in both UK clinical practice and published estimates [15,16,18,56], a service evaluation and national survey were conducted to obtain the models of anticoagulation, quantify its distribution and collect resource-use data to ensure that the analysis represented clinical practice [51]. Local care pathways will determine whether such savings can be realized consistently. However, several UK institutions, including some London hospitals and the Bradford and Sheffield NHS trusts, have already incorporated rivaroxaban into their standard VTE treatment algorithms. Our data will be relevant to clinicians in discussions at formulary committee meetings and to inform the structure of VTE care at the local level.

Inclusion of a disutility value for warfarin has further been applied in the model base case to account for patient preference. Although, the evidence is not strong, it is consistent, in that the significant patient burden associated with the use of warfarin and monitoring has been widely acknowledged. There is a degree of controversy over whether this quality of life disutility is ‘health-related’ and, therefore, should be appropriately considered from a health service perspective. We have taken the view that patient preference should be considered in the model base case, not least because there are aspects driving patient preference, that impact on health-related quality of life, including anxiety and uncertainty associated with staying in INR range. In addition, monitoring may have limitations for patients through the requirement for regular clinic attendance. Satisfaction with anticoagulation was found to be higher with rivaroxaban compared with LMWH/VKA in both the EINSTEIN DVT and EINSTEIN PE trials, as measured using the Anti-Clot Treatment Satisfaction (ACTS) scale. The ACTS questionnaire captures the patient perspective of burdens and benefits of anticoagulation, including health-related quality of life elements, such as confidence, reassurance and impact of bleeding and bruising on daily activities [49,50].

A limitation of using clinical study data to extrapolate to a real-life scenario is that the population and or disease management schedules may not be representative of daily care. However, the open-label design of EINSTEIN DVT and EINSTEIN PE, together with the limited exclusion criteria, flexibility of treatment duration, and availability of INR monitoring outside trial centres, allowed a broad and representative population to participate in the trials with a comparator relevant for the UK setting. As with any trial or cohort study, the centres involved in recruiting patients have expertise and interest in VTE treatment, and thus might be expected to provide a higher than usual standard of care (for instance, in maintaining higher time in INR target range).

Since rivaroxaban received marketing authorization for the treatment of VTE, three anticoagulants have received approval, apixaban, dabigatran and edoxaban, with all three demonstrating similar efficacy compared with the standard of care. In the UK setting, the NICE and the Scottish Medicines Consortium have accepted the cost-effectiveness of dabigatran in comparison with VKA, with apixaban being currently under assessment by both bodies. Determining the relative cost-effectiveness in the absence of direct head-to-head trials would depend on indirect comparisons of the trial results, which can only be considered as hypothesis-generating owing to differences in trial design (open-label versus double-blind), inclusion/exclusion criteria and the regional settings of the trials. Even then, residual bias or confounding will remain; therefore, indirect comparison has not been pursued. Furthermore, any differences in real-life cost-effectiveness of the novel oral anticoagulant regimens may critically depend on performance in real-life. This may include the ability of each novel regimen to displace initial LMWH therapy, supporting further out-of-hospital treatment, as well as patient acceptance and persistence on therapy, all of which are best assessed in real-life observational studies.

A scenario regarding treatment durations beyond those examined in EINSTEIN DVT or EINSTEIN PE was included in the analyses. This required assumptions regarding the long-term effectiveness of rivaroxaban. Furthermore, only limited trial data were available on the risk of VTE among patients treated with VKA for beyond 12 months. The data used in the model for the lifelong treatment scenario were drawn from a meta-analysis of three clinical trials [61]. However, the heterogeneity among these studies is acknowledged and highlights the continuing need for further evidence regarding the benefit–risk assessment of patients requiring long-term anticoagulation.

A main strength of this economic evaluation lies in the comprehensive model structure fed by robust clinical trials and extensive research to populate it. The model was developed over the course of the EINSTEIN studies in consultation with UK clinical and health economic experts, ensuring that the model clinical pathway was in line with UK clinical practice. Furthermore, sensitivity analyses have demonstrated the robustness of the findings.

These economic evaluations explored the long-term health and cost benefits of rivaroxaban compared with standard care in the treatment of patients with VTE based on the findings of EINSTEIN DVT and EINSTEIN PE [25,26]. Data from these studies provide further support for a shift in care in the treatment of VTE, which may no longer call for initial parenteral anticoagulation in some patients with DVT or PE.

## Conclusion

Based on data from the EINSTEIN studies and known local care pathways, this analysis suggests that rivaroxaban represents a cost-effective choice for acute treatment of DVT and PE and secondary prevention of VTE in the UK, compared with LMWH/VKA treatment, regardless of the required treatment duration.

## Additional files

Additional file 1: Figure S1. Tornado diagram of net monetary benefit of rivaroxaban versus LMWH/VKA for the treatment of DVT. Patients requiring 3 months **(a)**, 6 months **(b)**, 12 months **(c)** and lifelong anticoagulation **(d)**. DVT, deep vein thrombosis; GP, general practitioner; HR, hazard ratio; LMWH, low molecular weight heparin; OP, outpatient; OWSA, one-way sensitivity analysis; PE, pulmonary embolism; QALY, quality-adjusted life-year; VKA, vitamin K antagonist; VTE, venous thromboembolism. Additional file 2: Figure S2. Tornado diagram of net monetary benefit of rivaroxaban versus LMWH/VKA for the treatment of PE. Patients requiring 3 months **(a)**, 6 months **(b)**, 12 months **(c)** and lifelong anticoagulation **(d)**. EC, extra-cranial; GP, general practitioner; HR, hazard ratio; LMWH, low molecular weight heparin; OP, outpatient; OWSA, one-way sensitivity analysis; PE, pulmonary embolism; QALY, quality-adjusted life-year; VKA, vitamin K antagonist; VTE, venous thromboembolism; WARF, weighted average rating factor.
