# Supplementary material for: Cost-effectiveness analysis of treatment of venous thromboembolism with rivaroxaban compared with combined low molecular weight heparin/vitamin K antagonist
Source: Thromb J. 2015 Jun 11;13:20. doi: 10.1186/s12959-015-0051-3 (PMC4464718; doi:10.1186/s12959-015-0051-3)
Supplement: Additional file 2: Figure S2. — Tornado diagram of net monetary benefit of rivaroxaban versus LMWH/VKA for the treatment of PE. Patients requiring 3 months (a), 6 months (b), 12 months (c) and lifelong anticoagulation (d). EC, extra-cranial; GP, general practitioner; HR, hazard ratio; LMWH, low molecular weight heparin; OP, outpatient; OWSA, one-way sensitivity analysis; PE, pulmonary embolism; QALY, quality-adjusted life-year; VKA, vitamin K antagonist; VTE, venous thromboembolism; WARF, weighted average rating factor. [file 12959_2015_51_MOESM2_ESM.pdf]

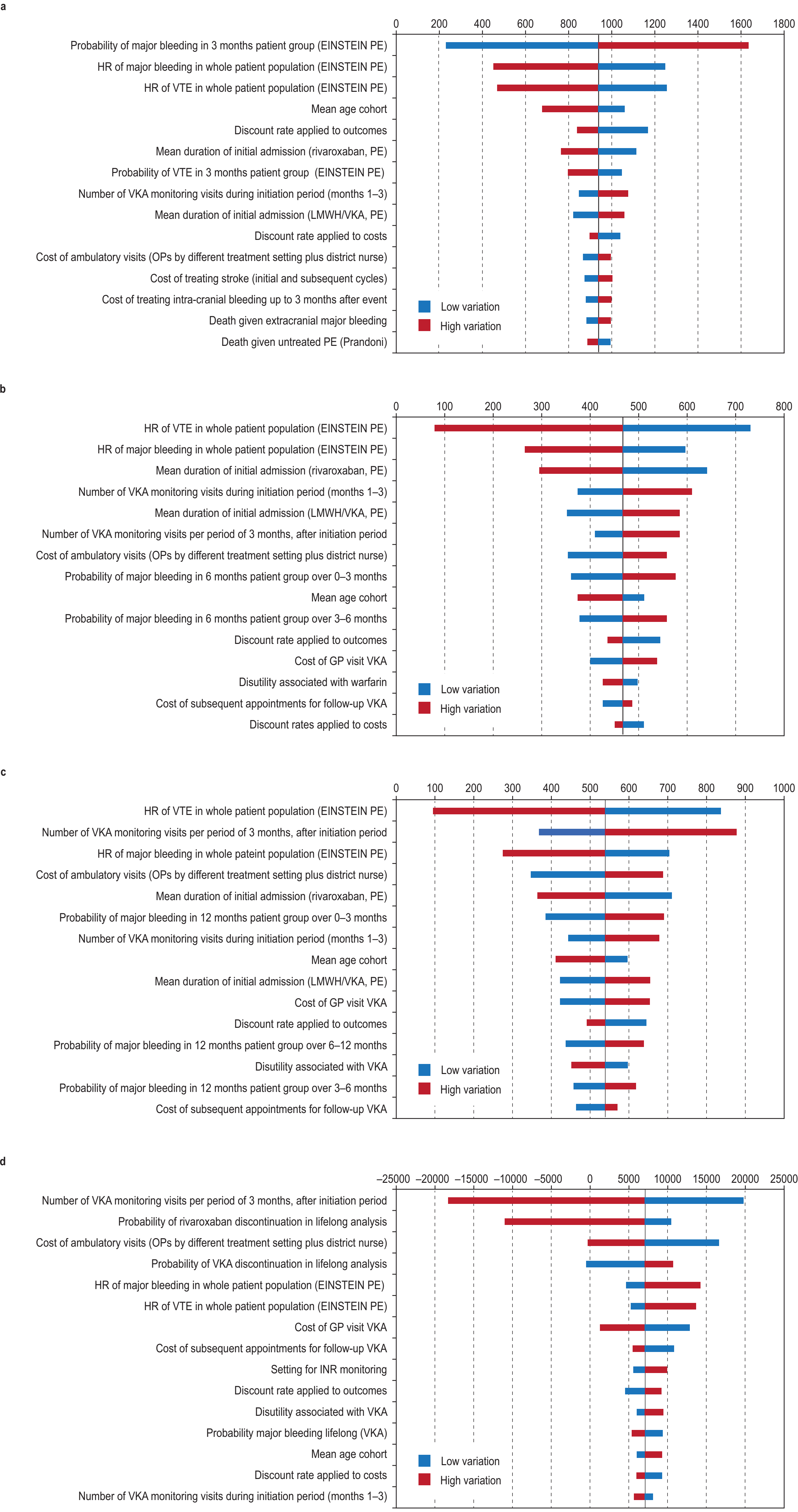

Figure S2 OWSA Tornado diagram of net monetary benefit of rivaroxaban versus LMWH/VKA for the treatment of PE – QALY based: 1 vs 2 (lifetime). Patients requiring 3 months (a), 6 months (b), 12 months (c), and lifelong anticoagulation (d).
GP, general practitioner; HR, hazard ratio; LMWH, low molecular weight heparin; OP, outpatient; OWSA, one-way sensitivity analysis; PE, pulmonary embolism; QALY, quality-adjusted life-year; VKA, vitamin K antagonist; VTE, venous thromboembolism.
